# Antibody-mediated immunity across the ABO barrier: natural isoagglutinins, effector injury, accommodation, and targeted intervention

**DOI:** 10.3389/fimmu.2026.1992914

**Published:** 2026-10-26

**Authors:** Dechuan Liu, Si Liu, Chenglong Yang, Xingyu Wu, Gang Wang

**Affiliations:** 1 Department of Donation Intensive Care Unit, The First Hospital of Jilin University, Changchun, China; 2 Department of Urology, The First Hospital of Jilin University, Changchun, China

**Keywords:** ABO-incompatible kidney transplantation, antibody-mediated immunity, complement, isoagglutinin, memory B cell, plasma cell

## Abstract

ABO-incompatible kidney transplantation (ABOi-KT) provides a clinical setting in which antibody-mediated injury and graft accommodation can be examined. Naturally occurring anti-A/B IgM and IgG differ in distribution and effector activity. After binding to graft endothelial glycans, these antibodies may activate complement, Fc-receptor-bearing cells, platelets, and coagulation, contributing to outcomes ranging from subclinical microvascular changes to thrombotic microangiopathy and antibody-mediated rejection. Although ABO and HLA antibodies can converge on complement- and Fc-mediated microvascular injury, ABO antigens are variably expressed terminal glycans whereas HLA antigens are polymorphic proteins; accordingly, persistent anti-A/B after accommodation may be clinically silent, while persistent HLA donor-specific antibody continues to signal potential ongoing alloimmune risk. Yet the same antibody specificity may later coexist with a functioning graft through accommodation, a dynamic state involving complement regulation, cytoprotective endothelial programs, altered antibody -glycan interactions, and partial downregulation of donor-specific antibody output. This Review integrates mechanistic and clinical evidence through August 2026 and maps each therapeutic intervention to its principal immune target: apheresis removes circulating antibody; rituximab depletes CD20-positive B cells but spares mature plasma cells; CD38- or proteasome-directed agents reduce antibody-secreting cells; and complement inhibitors interrupt downstream effector injury without eliminating antibody or its source. Conventional titers are method-dependent and do not capture all features relevant to antibody pathogenicity, including subclass, fine specificity, and complement-fixing activity. Functional assays may add information, but they require standardization and prospective validation before routine use. Low-titer protocols should therefore be evaluated prospectively, while intensified treatment for high-titer recipients should be guided by center capacity, prespecified safety limits, and clinical and pathological findings. Accommodation also remains incompletely defined and may reflect different combinations of graft adaptation and changes in antibody pathogenicity. Evidence for plasma-cell-directed therapy is explicitly hierarchical: ABO-specific support is confined to isolated rescue experience, whereas prospective anti-CD38 and controlled proteasome-inhibitor data derive from HLA sensitization or HLA-related ABMR and cannot establish routine efficacy in ABOi-KT.

## 1 Introduction

Kidney transplantation provides superior survival and quality of life compared with long-term dialysis, but access remains limited by organ scarcity (1, 2). For a candidate with a willing living donor, ABO blood-group incompatibility is a frequent barrier. Direct ABO-incompatible kidney transplantation (ABOi-KT) can shorten dialysis exposure and may confer a survival advantage over continued waiting when access to a compatible graft is delayed (3, 4).

ABOi-KT is also a distinctive model of antibody-mediated immunity in transplantation. Pre-existing anti-A/B antibodies recognize carbohydrate antigens on donor endothelium, can recruit complement and Fc-receptor-bearing effector cells, and can produce microvascular injury, thrombosis, and graft loss. Unlike HLA-directed donor-specific antibodies (DSA), however, anti-A/B antibodies target shared glycan structures rather than polymorphic protein alloantigens, and their persistence can coexist with stable graft function through accommodation. These distinctions make ABOi-KT a useful model for separating antibody abundance from pathogenic effect.

Terminology in this Review is deliberately restricted. Antibody-mediated immunity is the umbrella term for antibody production, circulating antibody, and complement-, Fc-receptor-, endothelial-, platelet-, and coagulation-mediated effects. Antibody-mediated alloimmunity is reserved for donor-directed responses against polymorphic alloantigens, principally HLA. Anti-A/B antibodies are naturally acquired, glycan-reactive antibodies that can become graft-reactive across an ABO barrier but are not synonymous with HLA alloantibodies. We therefore use antibody-mediated immunity consistently and reserve alloimmunity for responses to donor alloantigens.

Current protocols combine temporary antibody removal, control of antibody regeneration, and conventional peri-transplant immunosuppression. Patient and graft survival are excellent, yet comparative studies still identify additional early antibody-mediated rejection (ABMR), infection, and bleeding relative to ABO-compatible living donation (5, 6). Protocols also differ in titration method, target titer, apheresis modality, rituximab exposure, and surveillance (7, 8). A threshold validated in one center therefore cannot be assumed to represent the same antibody burden or biological risk elsewhere.

This Review reframes ABOi-KT around the immune compartments that produce, convey, and execute antibody-mediated injury (**Table 1**). It then uses those mechanisms to evaluate the principal clinical controversies: how titers should be interpreted, whether apparently low-risk recipients can safely avoid apheresis, when high-titer transplantation remains proportionate, and how post-transplant antibody rebound should influence treatment. The objective is a bridge between transplant immunology and an assay-aware, risk-adapted clinical pathway.

**Table 1:** Compartments of antibody-mediated immunity, pathogenic functions, and matched therapeutic targets.

Immune layer	Pathogenic function	Principal intervention	Mechanistic limitation	Clinical implication
Natural and memory B cells	Maintain repertoire, antigen presentation, recall, and plasmablast replenishment	Rituximab (CD20)	Depletes most CD20-positive cells but not mature plasma cells; regulatory B cells are also affected	Peripheral depletion does not prove elimination of antigen-experienced B-cell memory
Plasmablasts and long-lived plasma cells	Sustain or rapidly renew anti-A/B secretion	Anti-CD38 or proteasome-directed therapy	ABOi evidence is limited to isolated mixed-incompatibility rescue reports; prospective anti-CD38 and controlled proteasome-inhibitor data derive from HLA sensitization or HLA-related ABMR	Do not infer ABO-specific efficacy from target engagement or HLA-derived data; reserve for exceptional refractory production or prospective trials
Circulating IgM anti-A/B	High-avidity intravascular binding and efficient C1q recruitment	TPE, DFPP, or antigen-specific IA	Redistribution and renewed production follow removal; semi-selective IA may remove IgM less efficiently	Measure IgM separately and follow inter-session kinetics
Circulating IgG anti-A/B	Tissue distribution, complement activation, and Fcγ-receptor engagement	TPE or IA; IVIG as replacement/immunomodulation	Titer does not capture subclass, fine specificity, avidity, or complement-fixing potential	Pair same-method IgG kinetics with clinical and functional information
Complement cascade	C3/C5 amplification, anaphylatoxins, opsonization, and C5b-9-mediated endothelial stress	IVIG; C5 or other pathway inhibition in selected phenotypes	Does not remove antibody, Fc signaling, or the cellular source; encapsulated-bacterial infection risk	Use for demonstrated complement-dominant injury, not isolated C4d
Endothelium, Fc-dependent cells, platelets, and coagulation	Microvascular inflammation, TMA, and graft dysfunction	Biopsy-guided combination therapy; optimize maintenance immunosuppression	Several convergent pathways may persist when one effector arm is blocked	Treat the clinicopathologic ABMR phenotype rather than titer alone
Graft accommodation	Cytoprotection and complement regulation despite persistent or recurrent antibody	No validated accommodation-inducing drug; controlled early immune environment	Dynamic and potentially incomplete; not deletional tolerance	Require stable function and non-destructive pathology

ABMR, antibody-mediated rejection; DFPP, double-filtration plasmapheresis; IA, immunoadsorption; IVIG, intravenous immunoglobulin; TPE, therapeutic plasma exchange.

## 2 Review approach and scope

A targeted PubMed/MEDLINE search was updated through 20 August 2026 using combinations of “ABO incompatible”, “kidney transplantation”, “antibody-mediated alloimmunity”, “isoagglutinin”, “B cell”, “plasma cell”, “complement”, “desensitization”, “plasmapheresis”, “immunoadsorption”, “rituximab”, “accommodation”, “antibody-mediated rejection”, and “outcomes”. Reference lists of major reviews, meta-analyses, registry reports, multicenter cohorts, and recent primary studies were screened for additional reports. Titles and abstracts were first assessed for relevance, followed by full-text appraisal of potentially eligible reports. Sources were retained when they directly informed at least one prespecified domain: anti-A/B antibody origin and phenotype; B-cell or plasma-cell biology; complement-, Fc-, or endothelial injury and ABMR; accommodation; antibody measurement or functional phenotyping; intervention mechanisms; or risk-adapted clinical outcomes and surveillance.

The primary scope is planned adult living-donor ABOi-KT. Pediatric transplantation, deceased-donor pathways, A2/A2B allocation, and ex vivo blood-group conversion were considered only where they defined immunologic boundaries or future directions. Eligible evidence included published or accepted clinical trials, comparative and observational studies, registry analyses, mechanistic human or experimental studies, and relevant consensus or pathology reports. Systematic reviews and meta-analyses were used for contextualization and citation mining, whereas primary studies were preferred for key mechanistic or clinical claims. Direct ABOi-KT evidence was prioritized; HLA-derived, other-organ, or experimental evidence was retained only when mechanistically necessary and was labeled as extrapolative. Case reports were retained only when they illustrated an emerging rescue strategy or mechanistically informative phenotype.

Duplicate or substantially overlapping reports, sources outside the prespecified domains, reports with insufficient methodological or outcome detail, conference-only abstracts without adequate full-text data, unavailable full texts, and unpublished clinical material were excluded. Because this Review was designed as a narrative synthesis rather than a systematic or scoping review, it was not preregistered, does not claim exhaustive retrieval, and does not apply formal risk-of-bias scoring or quantitative pooling. Evidence was weighted according to study design, directness to ABOi-KT, cohort size, multicenter representation, assay comparability, and linkage to biopsy-defined or clinically meaningful outcomes. **Table 2** summarizes the eligibility and evidence-selection framework.

**Table 2:** Eligibility and evidence-selection framework for the targeted narrative review.

Domain	Eligibility or handling rule
Review design and purpose	Targeted narrative synthesis integrating mechanistic and clinical evidence relevant to antibody-mediated immunity in ABO-incompatible kidney transplantation; not designed as a systematic or scoping review.
Information sources and cutoff	PubMed/MEDLINE updated through 20 August 2026, supplemented by backward reference screening of major reviews, meta-analyses, registry reports, multicenter cohorts, and recent primary studies.
Primary population and context	Planned adult living-donor ABO-incompatible kidney transplantation. Pediatric, deceased-donor, A2/A2B allocation, and ex vivo blood-group conversion studies were retained only when they defined biological boundaries or future directions.
Eligible concepts	Anti-A/B antibody origin, isotype, subclass, and cellular source; complement-, Fc-, and endothelial injury; ABMR; accommodation; antibody measurement and functional phenotyping; intervention mechanisms; surveillance; outcomes; and competing harms.
Eligible evidence types	Published or accepted clinical trials, comparative and observational studies, registry analyses, mechanistic human or experimental studies, and relevant consensus or pathology reports. Systematic reviews and meta-analyses were used for contextualization and citation mining.
Screening and selection	Titles and abstracts were assessed for direct relevance to the prespecified domains, followed by full-text appraisal of potentially eligible reports.
Evidence prioritization	Direct ABO-incompatible kidney-transplant evidence, comparative studies, multicenter or registry analyses, and clinically informative contemporary cohorts were prioritized. Primary evidence was preferred for key claims; HLA-derived or other transplant evidence was labeled as extrapolative.
Restricted inclusion	Case reports were retained only when they illustrated an emerging rescue strategy or a mechanistically informative phenotype. Non-kidney or experimental evidence was retained only when directly relevant to mechanism or boundary conditions.
Exclusion criteria	Duplicate or substantially overlapping reports, sources outside the prespecified domains, reports with insufficient methodological or outcome detail, conference-only abstracts without adequate full-text data, unavailable full texts, and unpublished clinical material.
Synthesis and appraisal	Narrative thematic synthesis without formal risk-of-bias scoring or quantitative pooling. Evidence was interpreted according to design, directness to ABO-incompatible transplantation, cohort size, multicenter representation, assay comparability, and consistency with biopsy-defined or clinically meaningful outcomes.

This framework reports the procedure used for the narrative Review and does not imply a preregistered, exhaustive systematic- or scoping-review protocol. ABMR, antibody-mediated rejection; ABOi-KT, ABO-incompatible kidney transplantation; HLA, human leukocyte antigen.

## 3 Natural anti-A/B antibodies and the cellular architecture of antibody production and memory

### 3.1 The ABO glycan landscape at the graft interface

A and B determinants are terminal carbohydrate structures synthesized by ABO glycosyltransferases on glycoproteins and glycolipids. In the kidney, these glycans are displayed on vascular and glomerular endothelium and on tubular epithelium, but their density and topologic accessibility vary with A1/A2/B phenotype, secretor status, tissue compartment, and inflammatory activation (9). The relevant target is therefore not simply the donor blood-group label; it is a spatially organized glycan field encountered by antibody under conditions of reperfusion and endothelial stress.

Fine specificity matters within this glycan field. ABH-glycan arrays have shown that anti-A/B sera contain antibodies to multiple type-specific and chain-length-dependent structures that are incompletely represented by reagent red cells. Donor-specific subtype reactivity can change after antibody removal and transplantation even when a conventional hemagglutination endpoint appears similar (10, 11). These findings provide a molecular explanation for part of the disconnect between red-cell titers and endothelial pathogenicity.

### 3.2 Why “natural” anti-A/B antibodies are acquired immune repertoires

Anti-A/B antibodies are called natural because they arise without transfusion, pregnancy, or transplantation, but they are not generated in an antigen-free vacuum. They appear after infancy as exposure to microbial and environmental glycans that mimic A/B structures shapes the available repertoire; experimental exposure to blood-group-active bacteria can increase human isoagglutinins (12). B-1-like and marginal-zone-like responses are plausible sources of rapidly produced, broadly reactive IgM, while conventional B-cell activation and T-cell help can support class switching, affinity maturation, and memory. The relative contribution of these routes in an individual recipient is not measured by a routine titer.

Recipient blood group modifies this repertoire. Group O individuals commonly carry both IgM and class-switched IgG anti-A/B, whereas non-O recipients often have a different isotype balance. Age, prior microbial exposure, transfusion, pregnancy, and previous transplantation may further alter clonality and recall potential. Thus, the term “preformed antibody” describes timing, not a single cellular origin or fixed functional phenotype.

### 3.3 IgM and IgG are not interchangeable measures of risk

Pentameric IgM is largely intravascular, combines high avidity with efficient C1q recruitment, and can produce strong agglutination despite relatively low monomeric affinity. IgG is smaller, distributes more readily into extravascular and tissue compartments, persists longer, and can engage both complement and Fcγ receptors. These properties influence apheresis kinetics: an immediate intravascular reduction may be followed by redistribution, while ongoing cellular production sustains later rebound.

IgG subclass adds another layer. IgG1 and IgG3 generally recruit C1q and Fc-dependent effectors more efficiently than IgG2 or IgG4, but anti-ABO repertoires are often dominated by IgG1 and IgG2 and vary substantially between recipients (13). A reported hyperacute rejection associated with persistent anti-A/B IgG1 illustrates biological plausibility but does not establish a universal subclass threshold (14). More recent data suggest that C1q-binding activity may add risk information beyond conventional IgG/IgM titers, although validation remains limited (15). The available subclass studies are nevertheless heterogeneous in ABO direction, assay platform, reagents, sampling time, treatment exposure, and clinical endpoint. No prospective multicenter study has established a clinically actionable anti-A/B subclass threshold; subclass profiling should therefore remain an adjunctive research measure rather than a stand-alone treatment trigger.

### 3.4 B cells, memory B cells, plasmablasts, and plasma cells

The antibody response spans anatomically and therapeutically distinct compartments. Naïve and innate-like B cells can enter early responses; memory B cells rapidly differentiate after renewed antigen encounter; short-lived plasmablasts generate a burst of antibody; and long-lived plasma cells maintain secretion from protected bone-marrow or tissue niches. Memory B cells preserve recall capacity even when serum antibody is low, whereas mature plasma cells are CD20-negative and can continue secretion despite apparently complete peripheral B-cell depletion (16).

Prospective phenotyping after ABO-incompatible transplantation showed prolonged rituximab-associated B-cell depletion, naïve-skewed repopulation, and associations between rejection, activated B cells, plasmablasts, and reduced regulatory B-cell subsets (17). Separate longitudinal work supports acquired downregulation of donor-specific anti-A/B production after transplantation (18). These observations argue against using peripheral CD19/20 counts, serum titer, or plasmablast frequency as interchangeable surrogates; each samples a different layer of the antibody-mediated immune response.

### 3.5 Antibody rebound is a composite biological signal

Post-apheresis rebound can reflect rapid redistribution from extravascular spaces, continued secretion by long-lived plasma cells, and renewed output from memory B-cell-derived plasmablasts. The timescales overlap but are not identical. Immediate post-procedure sampling mainly measures removal efficiency, whereas inter-session kinetics capture redistribution plus production; sustained post-transplant increases may reflect recall in the presence of graft antigen. Serial same-method IgM and IgG measurements are therefore more informative than a single baseline value, but they remain phenotypic readouts rather than direct measurements of the responsible clone.

## 4 The complement–endothelium axis and antibody-mediated rejection

### 4.1 Classical complement activation converts binding into inflammation

When clustered anti-A/B antibody binds endothelial glycans, C1q can initiate the classical pathway, followed by C4 and C2 cleavage, C3 convertase formation, C3 opsonization, and amplification toward C5. C3a and C5a recruit and activate innate immune cells, whereas C5b-9 can directly stress or injure endothelium. IgM geometry, IgG subclass, antibody density, antigen density, and local complement regulation jointly determine whether binding remains silent or becomes destructive (15, 19, 20).

Complement is not an all-or-none cascade. Upstream C4 cleavage can leave stable C4d on peritubular capillaries while downstream amplification and terminal-pathway injury are constrained. Conversely, Fc-dependent inflammation can persist when C4d is absent or C5 is blocked. A case-control study linking higher pretransplant anti-ABO IgG, C5b-9 staining, and rejection supports the concept of incomplete rather than absolute complement control in some grafts (21; **Figure 1**).

**Figure 1:**
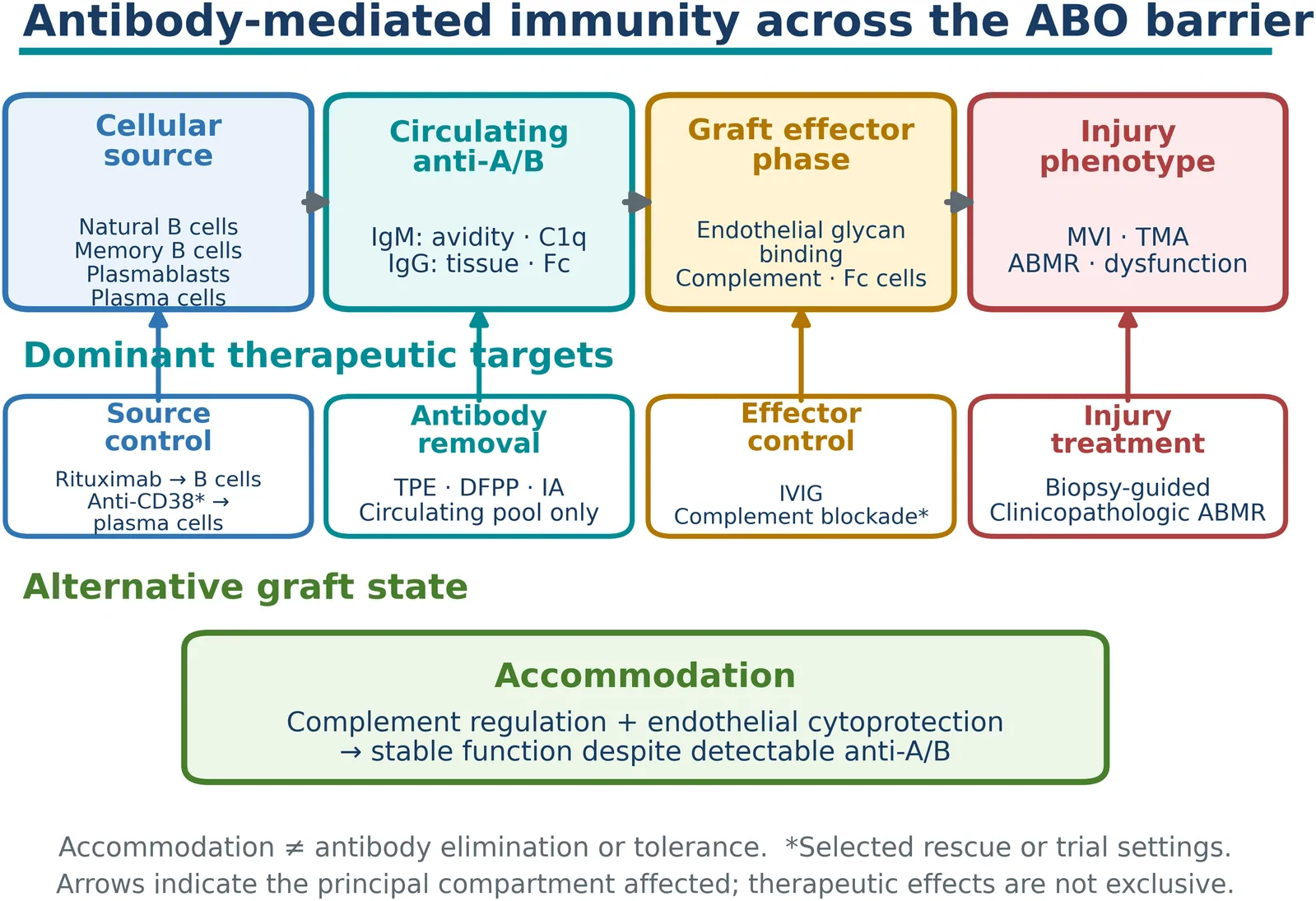
Cellular sources, effector pathways, and therapeutic targets within antibody-mediated immunity across the ABO barrier. The upper pathway is read from left to right: naïve or innate-like B cells and memory B cells generate plasmablasts and plasma cells; these compartments sustain circulating anti-A/B IgM and IgG; endothelial glycan binding then recruits classical complement and Fc-receptor-bearing cells, producing microvascular inflammation, thrombotic microangiopathy, antibody-mediated rejection, or graft dysfunction. IgM is predominantly intravascular and efficiently recruits C1q, whereas IgG distributes more readily into tissue and can engage both complement and Fc-dependent pathways. Vertical arrows identify the principal—not exclusive—compartment affected by each intervention: rituximab targets CD20-positive B cells but not mature plasma cells; anti-CD38 targets antibody-secreting cells; apheresis temporarily reduces the circulating antibody pool without erasing memory; intravenous immunoglobulin and complement inhibitors act mainly at downstream effector pathways; and biopsy-guided treatment responds to clinicopathologic injury. Accommodation is shown as an alternative graft state in which complement regulation and endothelial cytoprotection permit stable function despite detectable anti-A/B antibody; it does not imply antibody elimination or immunologic tolerance. Asterisks denote interventions supported mainly in selected rescue or trial settings rather than routine ABO-specific evidence. ABMR, antibody-mediated rejection; IVIG, intravenous immunoglobulin; TMA, thrombotic microangiopathy.

### 4.2 Endothelial activation links complement, Fc receptors, platelets, and coagulation

The graft endothelium is both the antigen-bearing target and an active immune organ. Sublytic complement and Fc-receptor-mediated interactions can induce a proadhesive, proinflammatory, and procoagulant state, recruiting monocytes, macrophages, natural killer cells, neutrophils, and platelets. Ischemia–reperfusion injury increases this susceptibility by perturbing the endothelial barrier and amplifying innate signals. The resulting continuum includes glomerulitis, peritubular capillaritis, thrombotic microangiopathy (TMA), cortical necrosis, and graft dysfunction (22, 23).

Concomitant HLA donor-specific antibody, delayed graft function, infection, and inadequate maintenance immunosuppression can lower the threshold at which anti-A/B binding becomes clinically apparent. Because ABO and HLA antibodies share an endothelial endpoint yet differ in target biology and the clinical meaning of post-transplant persistence, they should be evaluated together but interpreted separately.

### 4.3 ABO and HLA antibodies share an endothelial endpoint but not the same biology

ABO and HLA antibodies can converge on complement- and Fc-dependent microvascular inflammation, but the molecular targets that initiate injury are not interchangeable. ABO A/B determinants are terminal carbohydrate structures carried on endothelial glycoproteins and glycolipids, with density and accessibility shaped by donor subtype, secretor status, vascular bed, and inflammation (9–11). By contrast, donor HLA molecules are highly polymorphic protein alloantigens: class I is broadly expressed on nucleated cells and vascular endothelium, whereas class II is constitutive on professional antigen-presenting cells and can be induced on activated graft endothelium. Differences in target topology, inducibility, and epitope diversity influence antibody binding, complement recruitment, Fc-effector engagement, and the opportunity for ongoing T-cell-dependent reinforcement of the HLA response (22–25).

Accommodation is therefore not equally weighted in the two systems. In ABO-incompatible kidney transplantation, a clinically recognizable and often durable accommodated state may emerge after the early high-risk period: anti-A/B can persist or rebound, donor A/B antigen remains present, and peritubular capillary C4d may be detectable despite stable function and non-destructive histology (21, 26, 27). An analogous antibody-positive but functionally quiescent state can occur with HLA donor-specific antibody (DSA), but it is less predictable, less consistently durable, and lacks a validated clinical definition. Persistent or *de novo* HLA DSA—particularly when accompanied by complement-binding capacity, microvascular inflammation, or evolving transplant glomerulopathy—remains associated with chronic ABMR and graft loss (28–31). This is a difference in conditional risk, not an absolute rule: neither persistent anti-A/B nor persistent HLA DSA proves active ABMR without graft-level evidence.

Several nonexclusive mechanisms may explain why persistent anti-A/B is often better tolerated once ABO accommodation is established. The graft can upregulate complement regulators and cytoprotective or anti-apoptotic programs, modify glycan density or accessibility, and uncouple antibody binding or C4 cleavage from terminal-complement and cell-death pathways; recipient-side anti-A/B output may also fall selectively over time (18, 23, 25, 28, 32). By contrast, HLA proteins remain on graft endothelium and may be upregulated by inflammation, while class-switched HLA-specific memory B cells and long-lived plasma cells can be sustained by cognate T-cell help, affinity maturation, and epitope spreading. Thus, a stable accommodated ABO-incompatible graft should not be treated solely because anti-A/B or C4d persists, whereas persistent or newly detected HLA DSA should prompt longitudinal evaluation of graft function, proteinuria, antibody features, and—when clinically indicated—histologic or molecular evidence. The decision threshold in both settings is a clinicopathologic injury phenotype, not serology alone.

### 4.4 ABMR is a clinicopathologic phenotype, not a titer diagnosis

Active antibody-mediated rejection (ABMR) should be diagnosed by integrating graft function, microvascular morphology, serologic evidence, C4d, and—where validated—molecular information under current Banff principles (24, 33). In ABO-incompatible grafts, diffuse C4d can occur with stable function and little destructive injury, whereas C4d-negative ABMR remains possible (26). In this Review, a treatment-requiring syndrome is a clinicopathologically concordant episode: usually new or otherwise unexplained graft dysfunction (rising serum creatinine, reduced urine output, and/or new or worsening proteinuria) together with a biopsy meeting current Banff criteria for active or chronic active ABMR, after plausible mimics have been excluded. Active tissue injury may comprise microvascular inflammation, intimal or transmural arteritis, or acute thrombotic microangiopathy and should be interpreted with evidence of antibody-endothelium interaction and circulating donor-reactive antibody when the assay is sufficiently validated. Functional decline is supportive but not obligatory, because a protocol biopsy can disclose subclinical Banff-defined ABMR; these cases require individualized multidisciplinary assessment. Isolated titer rebound, isolated C4d staining, or donor-reactive antibody positivity without graft-level injury does not by itself define a treatment-requiring syndrome.

### 4.5 Functional phenotyping should complement, not merely replace, titers

The mechanistically relevant exposure includes antibody amount, isotype, subclass, avidity, fine glycan specificity, complement-fixing ability, and Fc-effector potential. C1q-binding, IgG-subclass, glycan-based, and endothelial-cell assays interrogate different parts of this biology, but none directly measures the complete graft-level injury phenotype. Available evidence remains limited by small or single-center studies and heterogeneous platforms (10, 11, 13–15, 25, 34, 35). These assays should therefore refine research phenotyping or selected adjunctive interpretation rather than replace same-method IgG/IgM titers, HLA risk assessment, graft-function surveillance, or biopsy. Clinical adoption requires evidence of analytical validity, cross-laboratory reproducibility, actionable turnaround time, acceptable cost, and prospective incremental value for biopsy-defined injury.

## 5 Accommodation as active graft adaptation

### 5.1 Defining accommodation without confusing it with tolerance

Accommodation is a state in which graft structure and function are preserved despite circulating antibodies directed against antigens expressed by the graft. Early human studies operationalized this state using detectable anti-A/B antibody, maintained graft antigen, satisfactory function, and non-destructive histology (27). It is not deletional tolerance: the recipient may retain antibody and antigen-experienced B-cell memory, and the graft may show biochemical evidence of antibody engagement.

### 5.2 Graft-intrinsic protection is multilayered

Proposed graft mechanisms include increased complement regulation (for example CD55, CD59, and related membrane regulators), anti-apoptotic and antioxidant programs, heme-oxygenase and stress-response pathways, altered endothelial signaling, and reduced propagation from sublytic complement to cell death. Anti-A/B ligation can generate signaling patterns distinct from HLA antibody ligation in endothelial models (25), and large-animal work has linked accommodation with CD59 upregulation (32). These findings are consistent with active cytoprotection, but they do not establish causality, and no single molecule is a validated clinical marker or sufficient explanation.

Human transcriptomic studies likewise suggest that accommodated ABO-incompatible grafts are molecularly distinct from HLA-incompatible grafts with subclinical ABMR (28). Earlier protocol-biopsy work identified altered inflammatory and protective gene expression rather than simple absence of immune activity (27). Sample sizes are small, definitions vary, and biopsies provide snapshots; accommodation should therefore be treated as a systems phenotype rather than a fixed transcriptional signature.

### 5.3 Recipient-side adaptation can reduce effective antibody pressure

Accommodation may be reinforced by changes outside the graft. Donor-specific anti-A/B production can decline over time despite preservation of responses to third-party blood-group antigens, consistent with selective downregulation rather than global impairment of antibody production (18). Changes in isotype, affinity, glycan-subtype recognition, antibody adsorption, and B-cell regulation may also reduce effective pathogenic pressure (10, 11). These processes need not eliminate measurable isoagglutinins. Because antibody quantity and quality can change while the graft is adapting, available studies cannot reliably apportion protection between graft-intrinsic resistance and reduced effective antibody pathogenicity.

### 5.4 Accommodation is dynamic and potentially incomplete

The early post-reperfusion period is a biologic bridge during which reduced antibody burden and adequate maintenance immunosuppression limit injury while graft resistance emerges. Infection, ischemia, nonadherence, HLA alloimmunity, or abrupt antibody amplification may destabilize the equilibrium. Persistent C4d or even downstream complement activity can coexist with acceptable function, raising the possibility of partial accommodation (21–23). Stable function and absence of destructive pathology remain the clinically meaningful evidence; elapsed time or C4d alone does not prove accommodation. Compared with the relatively well-recognized ABO phenotype, an analogous HLA DSA-positive state is less predictable and may not neutralize long-term risk; stable function at one time point should not be taken as durable HLA accommodation (28–31). The term may therefore encompass partial, transient, and context-dependent states rather than a single binary condition.

### 5.5 Accommodation remains an unresolved and potentially heterogeneous construct

No universally accepted biomarker or diagnostic test currently defines accommodation. Stable graft function, preserved histology, circulating anti-A/B antibody, C4d deposition, complement products, and proposed endothelial signatures each capture only part of the phenotype. Most human evidence derives from small cohorts, protocol-biopsy series, and cross-sectional molecular studies with heterogeneous definitions and sampling times (21–23, 27, 28). These data cannot determine whether an apparently stable graft is durably accommodated or has simply been observed during a low-injury interval.

Accommodation may therefore be an umbrella term for multiple biological states that differ in antibody exposure, complement activation, endothelial cytoprotection, tissue repair, and residual microvascular injury. The relative contributions of graft-intrinsic adaptation versus changes in antibody quantity, isotype, affinity, glycan specificity, and complement-fixing capacity remain unresolved because both sides evolve concurrently and are measured incompletely (10, 11, 18, 25, 28, 32). Longitudinal studies pairing standardized antibody phenotyping with serial tissue, complement, and endothelial-response measures are needed before accommodation can be assigned a causal biomarker or treated as a binary endpoint.

## 6 Measuring and phenotyping antibody-mediated immune activity

### 6.1 Why titers are not universal biological thresholds

Conventional tube testing, column agglutination, gel-card methods, automated platforms, reagent cell source, incubation conditions, endpoint grading, and dithiothreitol treatment can each change the reported anti-A/B titer. A multicenter United Kingdom study demonstrated clinically important interlaboratory variability, and contemporary laboratory comparisons confirm that apparently identical values may not be interchangeable (34–36). Serial samples are most interpretable when tested by one validated method in the same laboratory, with internal controls and a documented definition of meaningful change.

### 6.2 Antibody quality and kinetics matter

An endpoint titer collapses multiple biological features into one number. IgG and IgM should be distinguished when the method permits, because removal efficiency and effector potential differ. Baseline level, reduction per apheresis session, inter-session rebound, and stability immediately before transplantation describe a trajectory rather than a snapshot. Functional complement-binding, subclass, avidity, and glycan-specific assays may eventually refine risk, but prospective validation against biopsy-defined outcomes is still insufficient (34, 35).

The relevant question for a new biomarker is incremental discrimination. A complement-binding or subclass assay is clinically useful only if it predicts ABMR or apheresis response beyond the local IgG/IgM titer, HLA DSA, graft function, and treatment history. Analytical reproducibility, calibration material, turnaround time, and robustness after IVIG or apheresis are equally important. Without those data, a more elaborate assay may add complexity without reducing uncertainty. Mechanistic biomarker studies should prespecify sampling times and distinguish prediction of early injury, prediction of rebound, and identification of accommodation, because these are different clinical tasks.

### 6.3 Functional antibody assays remain implementation-limited

C1q-binding assays offer a functional readout of complement-recruiting capacity, but the signal depends on antibody concentration, the IgM/IgG and IgG-subclass mixture, complement source, incubation conditions, platform design, and positivity threshold. Signal saturation or interference can further complicate interpretation. The absence of a common reference material and clinically validated cutoff limits inter-laboratory transportability. A positive result shows C1q binding under assay conditions; it does not by itself establish complement-mediated graft injury or identify a patient who will benefit from complement blockade (15, 34, 35).

IgG-subclass profiling provides mechanistic granularity, yet results are sensitive to reagent specificity, calibration, lower limits of quantification, and differences in detection efficiency among subclasses. Interpretation is further complicated by mixtures of subclasses and by avidity, Fc glycosylation, epitope density, and concurrent IgM, none of which is captured by subclass concentration alone. No prospectively validated subclass threshold currently defines the need for apheresis, plasma-cell-directed therapy, or complement inhibition in ABO-incompatible kidney transplantation (13–15).

Glycan-based assays can resolve fine specificity that conventional red-cell agglutination misses, but array composition, glycan purity, valency, orientation, linker chemistry, surface density, and detection method all influence binding. A synthetic glycan surface also cannot fully reproduce the topology, membrane mobility, accessory molecules, or inflammatory state of graft endothelium. These platforms require specialized reagents and analytical expertise, remain vulnerable to batch effects, and may be too costly or slow for time-sensitive peri-transplant decisions (10, 11).

Endothelial-cell assays are closer to the biological target but introduce another layer of variability. Cell source, ABO subtype and secretor phenotype, passage number, culture and activation conditions, baseline antigen density, and the presence of complement or Fc-receptor-bearing effector cells can materially alter the readout. Live-cell infrastructure, operator expertise, batch-to-batch variability, cost, and prolonged turnaround complicate external quality assurance and multicenter implementation (25).

Before any of these assays enters routine practice, its intended use must be defined—for example, baseline risk stratification, prediction of rebound, biopsy triage, or identification of accommodation. Preanalytical conditions and analytical procedures should be locked; common calibrators, reference ranges, proficiency testing, and acceptable intra- and inter-laboratory variation should be established; performance after apheresis or IVIG should be characterized; and turnaround time and cost should fit the clinical decision. Most importantly, prospective multicenter studies must show incremental discrimination and clinical utility beyond local titers, HLA antibody assessment, graft function, and biopsy-defined outcomes. Until these requirements are met, functional antibody assays should be presented as research tools or selective adjuncts, not as stand-alone determinants of treatment.

### 6.4 Build a multidomain risk phenotype

The immunologic baseline should include donor and recipient ABO type and relevant subtype, same-laboratory IgG and IgM titers, HLA antibody testing and crossmatch, prior sensitizing events, and previous response kinetics. Group O recipients and those with a high IgG component often require more antibody removal. Historical transplant-day targets such as ≤1:8 or ≤1:16 remain useful operational anchors, but their meaning is method-specific (37). Clinical vulnerability—active infection, bleeding risk, vascular access, frailty, adherence, and tolerance of cumulative immunosuppression—must be considered alongside the antibody phenotype and the center’s capacity to provide apheresis, expert transplant pathology support, and rescue care.

### 6.5 The laboratory–clinical interface is part of the intervention

Reports should state the method, isotype handling, endpoint definition, reagent cells, and whether the result is directly comparable with prior samples. Programs should audit interoperator variation, participate in external quality assessment, retain reference material where feasible, and define how a one- or two-dilution change will affect treatment. This infrastructure is not merely technical: when a clinical pathway is driven by serial titers, assay uncertainty becomes a component of treatment uncertainty.

## 7 Matching interventions to compartments of antibody-mediated immunity

### 7.1 Separate the target compartments before combining therapies

A mechanism-based regimen distinguishes at least five targets: circulating antibody, CD20-positive B cells, antibody-secreting cells, complement/Fc-mediated effector injury, and the graft environment in which accommodation develops. No available therapy controls every layer. Combining agents is rational only when each component addresses a plausible source of risk; otherwise, added intensity mainly compounds infection, bleeding, and hypogammaglobulinemia.

### 7.2 Apheresis removes circulating antibody but not its cellular sources

Therapeutic plasma exchange (TPE), double-filtration plasmapheresis (DFPP), and immunoadsorption (IA) reduce the circulating antibody compartment. TPE is broad and also removes complement, fibrinogen, protective immunoglobulins, albumin-bound molecules, and administered biologics. DFPP preferentially retains larger proteins but remains incompletely selective. Semi-selective IA is generally more efficient for IgG than IgM, whereas antigen-specific IA directly captures anti-A/B antibodies (38–40). None eliminates memory B cells or long-lived plasma cells.

The apparent response depends on when and where it is measured. Immediate decline describes intravascular removal; inter-session rebound includes redistribution and new secretion. Choice among TPE, DFPP, and IA should therefore follow IgM/IgG pattern, required speed, response kinetics, coagulation status, access, local expertise, and cost rather than a universal modality hierarchy (41–44).

### 7.3 Rituximab targets CD20-positive B cells, not mature plasma cells

Rituximab depletes most circulating and lymphoid CD20-positive B cells, reducing antigen presentation and the pool available for memory activation and new plasmablast generation. Mature plasma cells lack CD20, so rituximab neither explains an immediate titer fall nor guarantees cessation of antibody secretion. Its effects are delayed and can persist through the period of B-cell reconstitution (17, 45, 46).

Dose and omission should be treated as protocol questions. Low-dose rituximab has been associated with acceptable outcomes in selected low-titer cohorts, whereas rituximab-free practice has shown inconsistent rejection risk (47, 48). B-cell depletion also removes protective and regulatory populations, impairs vaccine responses, and can contribute to neutropenia, hypogammaglobulinemia, and viral or bacterial infection.

### 7.4 Plasma-cell-directed therapies: target plausibility exceeds ABO-specific evidence

CD38-directed antibodies and proteasome inhibitors act closer to the source of ongoing secretion than rituximab, but mechanistic proximity should not be equated with disease-specific efficacy. Daratumumab can deplete or suppress antibody-secreting cells; however, CD38 is also expressed by plasmablasts, natural killer cells, and other immune populations. Proteasome inhibition exploits the secretory stress of plasma cells but is likewise not selective for anti-A/B-producing clones. The evidence should therefore be read in three tiers: direct ABOi-KT data, evidence from HLA sensitization or HLA-related ABMR, and mechanistic inference.

Direct ABOi-KT evidence is currently the weakest tier. Published use of daratumumab is limited to isolated rescue experience, including a recipient with combined ABO and HLA incompatibility treated with blood group-specific apheresis and other concomitant interventions (49). That report cannot isolate the effect of CD38 blockade, determine whether the relevant target was anti-A/B or HLA antibody production, or support prophylactic use. No prospective ABO-specific trial has established that daratumumab or a proteasome inhibitor improves biopsy-defined ABMR, graft survival, or patient-centered outcomes.

The next tier is indirect HLA-derived evidence. In a single-center phase 1/2 study of candidates with calculated panel-reactive antibody greater than 95%, daratumumab produced rapid but often transient reductions in anti-HLA antibodies, total immunoglobulins, vaccine antibodies, and natural isohemagglutinins; rebound and incomplete durability were prominent (50). The study was designed for HLA sensitization rather than planned ABOi-KT, so the fall in isohemagglutinins supports target engagement but not ABO-specific clinical efficacy. Proteasome-inhibitor experience is also dominated by HLA desensitization and HLA donor-specific-antibody-positive ABMR. In the randomized BORTEJECT trial, bortezomib did not prevent estimated glomerular filtration rate decline or meaningfully improve donor-specific antibody or biopsy outcomes in late ABMR and caused substantial toxicity (51). This negative HLA-ABMR result neither proves nor disproves efficacy in acute ABO-mediated injury, but it argues against assuming a class effect across antibody phenotypes.

Accordingly, evidence from HLA sensitization or HLA-related ABMR should be labeled as extrapolative whenever it is used to discuss ABOi-KT. Cytopenia, infection, neuropathy with proteasome inhibitors, prolonged hypogammaglobulinemia, loss of protective antibodies, memory-driven rebound, and interference with blood-bank testing after anti-CD38 treatment are material risks. Outside a trial, plasma-cell-directed therapy should be reserved for exceptional, biopsy-supported refractory phenotypes with evidence of persistent antibody production after conventional antibody removal and maintenance immunosuppression have been optimized; it should not be incorporated routinely into ABOi prophylaxis.

Because plasma-cell-directed rescue can deepen immunoparesis, it should not begin without a prespecified infection-prevention plan (52–57). Before the first dose, this plan should document a complete blood count with differential, quantitative IgG/IgA/IgM, vaccination status, hepatitis B surface antigen, anti-HBc, and anti-HBs (with HBV DNA when indicated) CMV risk or prior DNAemia, and BK polyomavirus DNAemia; active infection should be excluded. Trimethoprim-sulfamethoxazole prophylaxis against Pneumocystis jirovecii should be continued or reinstituted during augmented immunosuppression; acyclovir or valacyclovir should cover anti-CD38 or proteasome-inhibitor exposure and, for daratumumab, continue for 3 months after the final dose. Positive HBV markers require transplant-infectious-disease or hepatology review for nucleos(t)ide-analogue prophylaxis or HBV-DNA surveillance. CMV and BK quantitative PCR surveillance should be intensified, with CMV pre-emptive therapy and BK-directed immunosuppression adjustment according to transplant protocols. Routine antibacterial or antifungal prophylaxis is not justified solely by drug exposure and should be reserved for prolonged severe neutropenia or another defined epidemiologic risk. These safeguards are extrapolated from hematology and solid-organ-transplant practice rather than validated as an ABOi-KT-specific bundle.

### 7.5 IVIG and complement inhibitors act downstream of antibody production

Intravenous immunoglobulin (IVIG) replaces some removed IgG and can modulate Fc receptors, complement, and immune-cell signaling, but it does not selectively remove anti-A/B clones or abolish plasma-cell output (58). It should be timed after rather than immediately before a planned removal session. Hemolysis, thrombosis, volume load, and serologic interference remain relevant.

Complement-directed drugs interrupt effector injury after antibody has bound. Eculizumab blocks C5 cleavage and terminal-pathway activity but leaves antibody, C1q–C4 engagement, Fc-receptor signaling, and antibody production intact. Small ABOi-KT series support short-term rescue in selected high-titer or complement-dominant phenotypes (59); they do not establish routine prophylaxis. Target engagement should be demonstrated, and infection prevention—especially against encapsulated bacteria—is integral to the intervention.

### 7.6 Conventional immunosuppression limits cellular help while accommodation emerges

Tacrolimus, mycophenolic-acid therapy, corticosteroids, and induction do not remove preformed anti-A/B antibody. Their role in antibody-mediated immunity is to constrain T-cell help, B-cell activation, plasmablast differentiation, and parallel cellular rejection during the period of greatest endothelial vulnerability (60–62). Adequate exposure before reperfusion is therefore mechanistically important, but escalation cannot compensate indefinitely for uncontrolled antibody or active effector injury.

## 8 Clinical translation: low-titer de-escalation and high-titer intensification

### 8.1 Low baseline titers do not automatically justify omission of apheresis

Several centers have reported successful transplantation with reduced or no antibody removal when baseline titers are low (63, 64). These observations support formal de-escalation studies, but they do not establish a universal low-risk phenotype. In a 2026 French multicenter study restricted to recipients with baseline IgG titers ≤1:8, omission of pretransplant apheresis was associated with more rejection and lower three-year event-free survival than use of apheresis (79% versus 90%) (65). The excess rejection was predominantly antibody-mediated: ABMR occurred in 10 of 37 recipients without apheresis (27.0%) versus 4 of 41 with apheresis (9.8%), whereas TCMR occurred in 2 of 37 (5.4%) versus 1 of 41 (2.4%); borderline rejection occurred in 4 of 37 (10.8%) versus 1 of 41 (2.4%). Early ABMR occurred in 5 of 37 recipients without apheresis (13.5%) versus 2 of 41 with apheresis (4.9%), and four no-apheresis recipients developed hyperacute rejection with thrombotic microangiopathy, including one case of cortical necrosis with primary nonfunction. However, the ABMR comparison showed only a statistical trend (p = 0.075), and none of the subtype-specific comparisons reached significance. The small event counts, lack of central biopsy review, and between-center assay heterogeneity therefore support an ABMR-predominant signal rather than proof that apheresis selectively prevents ABMR. Residual confounding limits causal inference, yet the finding directly challenges the assumption that one low pretreatment value proves that antibody removal is unnecessary. De-escalation should be defined by the local assay, repeated values, kinetics, HLA risk, and prospective rescue rules. **Table 3** summarizes the assay, kinetic, HLA, patient, and center-level factors that should be considered in addition to baseline titer before protocol de-escalation.

**Table 3:** Factors beyond baseline titer that influence candidacy for protocol de-escalation.

Domain	More supportive of de-escalation	Argues against de-escalation	Minimum safeguard
Assay reliability	Repeated, concordant results from the same validated laboratory method	Method change, discordant results, or poorly characterized inter-laboratory variation	Repeat testing with the local validated IgM/IgG method before risk assignment
Antibody kinetics and isotype	Stable or falling values, favorable between-session kinetics, and no dominant IgG concern	Rapid rebound, rising trend, a substantial IgG component, or uncertain kinetics	Serial same-method IgM/IgG measurements with a prespecified escalation trigger
HLA immune context	No clinically relevant HLA DSA and an acceptable crossmatch	Relevant HLA DSA, positive or ambiguous crossmatch, or previous HLA-related ABMR	Dedicated HLA assessment; do not merge ABO and HLA risk into one titer category
ABO and immune history	No additional high-risk ABO direction, subtype, or sensitizing history	Group O recipient with prominent IgG, uncertain subtype, or history suggesting brisk recall	Interpret ABO direction, subtype, isotype, and sensitization history together
Clinical vulnerability	No active infection; acceptable immunoglobulin, coagulation, and adherence profile	Active infection, hypogammaglobulinemia, coagulopathy, frailty, or adherence concern	Multidisciplinary safety review before any protocol minimization
Center capability	Timely same-laboratory titers, rescue apheresis, expert pathology, and biopsy access	Delayed testing or rescue, limited pathology support, or inconsistent surveillance	Written monitoring, rescue, escalation, and stopping plan
Transplant readiness	Adequate immunosuppressive exposure, favorable kinetics, and no unresolved toxicity	Drug underexposure, unstable antibody course, active infection, or treatment toxicity	Final multidisciplinary readiness gate immediately before transplantation

No single factor establishes eligibility for de-escalation. Minimization should occur only within a locally validated pathway with prospective monitoring and immediate rescue capability. ABMR, antibody-mediated rejection; DSA, donor-specific antibody; IgG, immunoglobulin G; IgM, immunoglobulin M.

Several mechanisms could make a low baseline value falsely reassuring. The sample may have been generated by a less sensitive method, the IgG component may be masked by IgM handling, antibody with high functional activity may be present at a low agglutination endpoint, or memory responses may accelerate after graft exposure. There may also be center-level confounding: programs that safely minimize treatment often rely on tightly controlled laboratory procedures and rapid rescue capacity. A “no-apheresis” strategy should therefore be evaluated as a complete protocol with defined eligibility, not as a titer cutoff detached from its laboratory and clinical system.

### 8.2 High and very high titers: distinguish feasibility from proportional benefit

High baseline titers usually require more sessions and create more opportunities for coagulopathy, hypogammaglobulinemia, access complications, and infection. In a 2024 series of eight recipients with titers ≥1:512, patient and graft survival were 100% after a median of 13 apheresis sessions, but acute antibody-mediated rejection occurred in 37% and infection was frequent (66). A 2026 German multicenter survey confirmed that experienced programs will transplant selected very high-titer recipients, commonly with rituximab and antigen-specific immunoadsorption, but eligibility and stopping rules varied substantially (67). Longer-term data likewise suggest that survival can be preserved at the cost of greater treatment burden and, in some cohorts, more chronic active ABMR (68). High titer is therefore neither an absolute contraindication nor a mandate for unlimited escalation.

Published high-titer cohorts also contain an important selection effect: recipients who failed to reach the transplant-day target, developed toxicity, or were redirected to another pathway may be absent from outcome analyses. A center can report excellent post-transplant survival while the overall strategy still imposes substantial pretransplant burden. Denominator-based reporting should therefore include all patients who start desensitization, the proportion transplanted, time and procedures required, reasons for discontinuation, adverse events before surgery, and outcomes of alternative pathways.

### 8.3 Compare direct transplantation with compatible alternatives before treatment

Kidney-paired donation (KPD), another compatible living donor, and expected deceased-donor waiting time should be considered before the recipient is exposed to desensitization. KPD avoids ABO-directed therapy but may introduce clinically important delay and may be less successful for group O or HLA-sensitized candidates. Direct ABOi-KT is most defensible when it provides a timely transplant and the center can deliver standardized testing, apheresis, expert pathology, and rescue. This is a shared decision about competing pathways rather than a laboratory threshold alone.

### 8.4 Define transplant readiness and stopping rules prospectively

Readiness should require a reproducible titer at or below the center’s validated target, favorable rebound kinetics, therapeutic maintenance-drug exposure, acceptable coagulation and IgG status, no active infection, acceptable HLA crossmatch/DSA risk, and multidisciplinary agreement. The maximum acceptable treatment burden, toxicity limits, criteria for switching modalities, and conditions for deferring transplantation should be specified before the first procedure. Refractory rebound, uncontrolled infection, progressive hypogammaglobulinemia, coagulopathy, or loss of proportional benefit are valid reasons to pause. **Table 4** and **Figure 2** translate these principles into a risk-adapted pathway; the titer bands are illustrative and must be calibrated to the local method.

**Table 4:** Proposed assay-aware, risk-adapted desensitization framework.

Risk phenotype*	Assessment	Typical intensity	Readiness	Key caution
Low (example ≤1:8)	Confirm with repeat same-laboratory IgG/IgM; review ABO direction, DSA and prior kinetics	Pretransplant immunosuppression; center-specific rituximab. Do not routinely omit apheresis outside a validated protocol	Reproducible low/stable titer; acceptable coagulation/IgG; no active infection	The 2026 multicenter study found worse outcomes without apheresis despite low baseline titers (65)
Intermediate (example 1:16–1:64)	Repeat titer; quantify response and rebound; assess bleeding/infection vulnerability	Serial TPE or DFPP, or IA; rituximab commonly used; adjust after every 1–2 sessions	At/below validated center target (often ≤1:8) with favorable kinetics	Escalate, switch modality or postpone if plateau/rebound or toxicity occurs
High (example ≥1:128; very high ≥1:512)	Expert-center review; KPD comparison; dual ABO/HLA risk; prespecified resource and safety limits	Antigen-specific or semi-selective IA ± TPE/DFPP; rituximab; individualized rescue only if justified	Target achieved without unsafe treatment burden; multidisciplinary approval	Feasible in selected recipients, but treatment burden, ABMR and infection are high (66–68)

*Titer bands are examples only. A value such as 1:8 or 1:64 is meaningful only within the validated method that generated it. The framework is not a clinical guideline and requires center-specific validation. DSA, donor-specific antibody; DFPP, double-filtration plasmapheresis; IA, immunoadsorption; TPE, therapeutic plasma exchange.

**Figure 2:**
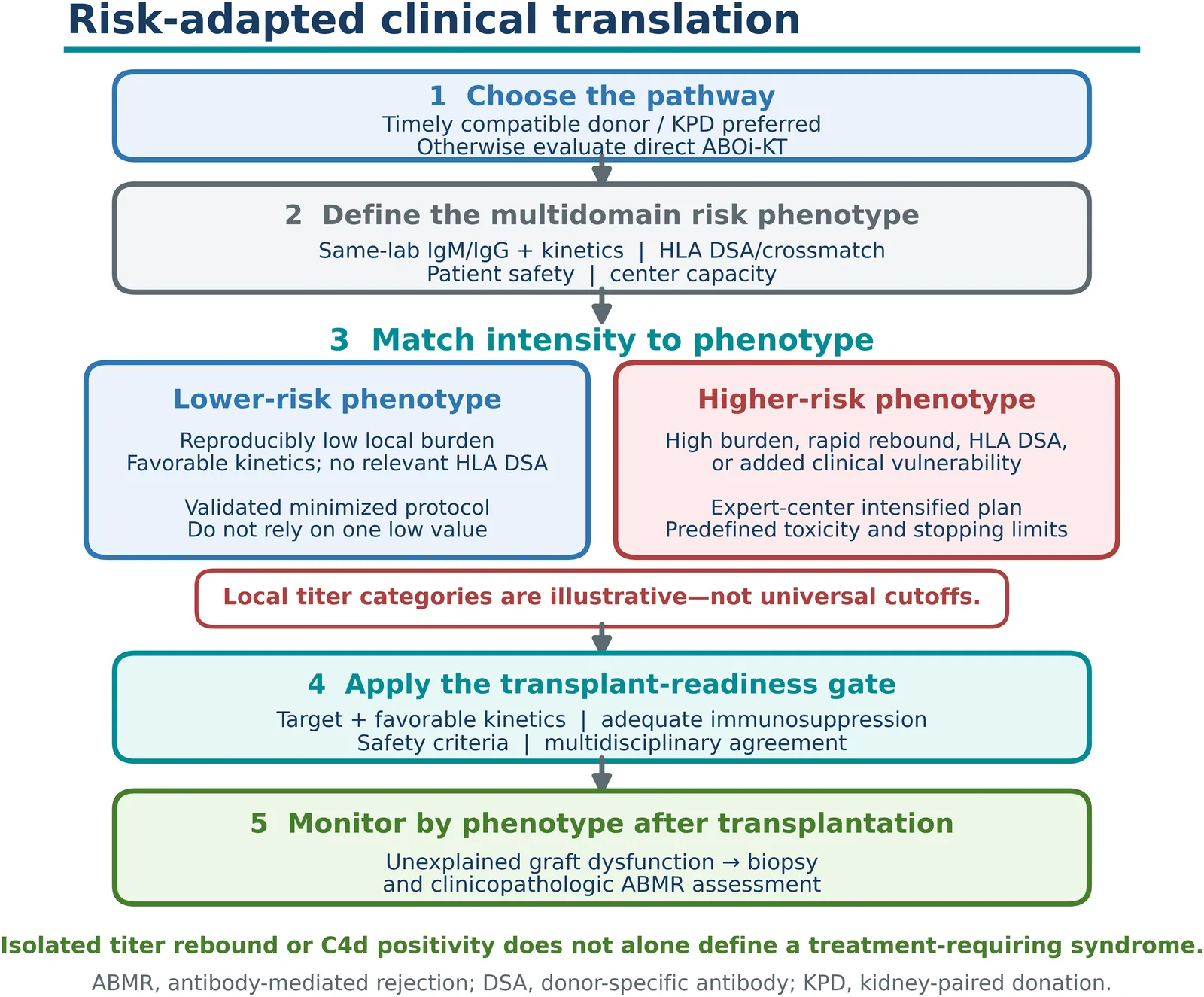
Assay-aware, risk-adapted pathway for planned ABO-incompatible kidney transplantation. Step 1 prioritizes a timely ABO-compatible donor or kidney-paired donation (KPD) when feasible before direct ABOi-KT. Step 2 defines a multidomain phenotype using serial same-laboratory IgM and IgG titers and kinetics, ABO direction or subtype, HLA donor-specific antibody (DSA) and crossmatch, patient safety, and center capacity. Step 3 matches treatment intensity to that phenotype: reproducibly low local antibody burden, favorable kinetics, and absence of clinically relevant HLA DSA may support a validated minimized protocol, whereas high burden, rapid rebound, HLA DSA, or clinical vulnerability supports an expert-center intensified plan with prespecified toxicity and stopping limits. Any local titer category is illustrative and cannot be transferred as a universal cutoff between laboratories. Step 4 applies a transplant-readiness gate requiring the locally validated antibody target and favorable kinetics, adequate immunosuppressive exposure, acceptable safety parameters, and multidisciplinary agreement. Step 5 monitors the post-transplant phenotype; unexplained graft dysfunction should prompt biopsy and clinicopathologic ABMR assessment. Isolated titer rebound or isolated C4d staining does not by itself establish a treatment-requiring syndrome. Arrows show the conceptual sequence and do not constitute a validated clinical algorithm. ABMR, antibody-mediated rejection; ABOi-KT, ABO-incompatible kidney transplantation; DSA, donor-specific antibody; KPD, kidney-paired donation.

## 9 Post-transplant surveillance and phenotype-based treatment

### 9.1 The early critical period

Immunologic risk is concentrated in the first one to two weeks, before accommodation is established. Early surveillance should combine graft function, urine output, blood count, tacrolimus exposure, and same-method anti-A/B titers; hemolysis/TMA markers and Doppler ultrasonography are added when clinically indicated. Monitoring intensity should reflect baseline titer, rebound kinetics, concomitant HLA DSA, delayed graft function, and cumulative treatment. **Table 5** links phases of care to specific decisions rather than presenting a universal schedule.

**Table 5:** Monitoring domains and linked clinical decisions.

Phase	Minimum monitoring domains	Linked decision
Before desensitization	ABO type/subtype; same-lab IgG/IgM titers; HLA DSA/crossmatch; CBC, fibrinogen/coagulation, IgG; infection screen	Confirm direct ABOi rationale; select method and intensity; vaccinate before rituximab when possible
During apheresis	Titer after every 1–2 sessions; CBC; fibrinogen/coagulation; albumin; IgG; access complications	Continue, switch or pause according to response, rebound and toxicity; time drugs after removal sessions
Day before/day of KT	Repeat titer by the same method; tacrolimus exposure; CBC/coagulation/fibrinogen; infection status	Proceed only if center target and safety criteria are met
Postoperative days 0–7	Daily graft function and titer; CBC/platelets; hemolysis/TMA markers when indicated; tacrolimus; Doppler as clinically indicated	Urgent biopsy for otherwise unexplained dysfunction. Treat a clinicopathologically concordant syndrome (graft dysfunction or, exceptionally, a high-risk subclinical biopsy plus Banff-defined active/chronic active ABMR after exclusion of mimics), not isolated titer rebound or C4d staining.
Weeks 2–4	Risk-spaced titers and renal function; CBC with differential and quantitative IgG at least monthly after plasma-cell-directed rescue; CMV and BK quantitative PCR according to intensified transplant surveillance	Continue HSV/VZV and Pneumocystis prophylaxis; use CMV pre-emptive therapy and BK-directed immunosuppression adjustment per protocol; consider Ig replacement for IgG <400 mg/dL with recurrent or severe infection
Months 1–12	Routine transplant surveillance; BK/CMV; proteinuria; DSA when clinically indicated; biopsy for dysfunction	Avoid treatment of isolated C4d; optimize maintenance immunosuppression and infection prevention

Monitoring should be intensified for high baseline titers, rapid rebound, concomitant HLA DSA, delayed graft function, TMA, or intensive rescue therapy. CBC, complete blood count; CMV, cytomegalovirus; DSA, donor-specific antibody; KT, kidney transplantation; TMA, thrombotic microangiopathy. Operational treatment threshold: a treatment-requiring syndrome is usually new or unexplained graft dysfunction plus biopsy-defined active or chronic active ABMR; Banff-defined subclinical ABMR is an exception requiring individualized review. Relevant tissue injury includes microvascular inflammation, arteritis, or acute TMA with evidence of antibody-endothelium interaction and validated donor-reactive antibody testing, after exclusion of competing causes. Isolated titer rebound, isolated C4d, or antibody positivity without tissue injury is insufficient.

### 9.2 Rebound is a signal for evaluation, not an automatic treatment trigger

A titer rise is most concerning when it is rapid, sustained, and accompanied by oliguria, creatinine elevation, proteinuria, thrombocytopenia, hemolysis, or other evidence of graft injury. Some programs resume antibody removal at a method-specific threshold, but clinical and pathologic context should dominate (34, 69). Treating an isolated laboratory rise can expose an accommodated recipient to unnecessary apheresis and immunosuppression. Conversely, unexplained dysfunction with rising antibody warrants urgent evaluation and usually allograft biopsy.

### 9.3 Treat biopsy-defined injury according to its effector phenotype

Suspected ABMR should be classified using current Banff principles (24). Treatment may include TPE or immunoadsorption, IVIG, corticosteroids, and optimization of maintenance immunosuppression. Rituximab, complement blockade, plasma-cell-directed therapy, or cytokine-pathway inhibition may be considered for selected refractory phenotypes after the target mechanism and infection risk are reviewed (49, 59). Persistent antibody, isolated C4d, and biopsy-defined microvascular injury are not interchangeable states and should not receive identical treatment. For plasma-cell-directed agents, direct ABO-specific support remains case-level; HLA desensitization or HLA-ABMR studies provide only indirect guidance.

### 9.4 Infection prevention should follow cumulative immune exposure

ABOi-KT recipients should receive standard cytomegalovirus, Pneumocystis, and other prophylaxis according to serostatus and total immunosuppression. Vaccination should precede rituximab when possible. BK polyomavirus and cytomegalovirus surveillance, quantitative immunoglobulin assessment after repeated apheresis or B-cell depletion, and prompt evaluation of recurrent bacterial infection are more informative than the ABO label alone. Prophylaxis and surveillance should be intensified when mechanistic layers of therapy accumulate. For plasma-cell-directed rescue, quantitative IgG should be measured before treatment, at least monthly during therapy, and for 3–6 months afterward or until recovery. Immunoglobulin replacement should not be automatic: it is most defensible when IgG is below 400 mg/dL with recurrent, severe, or life-threatening infection; some expert frameworks also consider IgG below 500 mg/dL with recurrent respiratory infection (52, 53). A practical starting regimen is IVIG 0.4–0.6 g/kg every 3–4 weeks, titrated to infection control and a trough generally above 400–500 mg/dL. Renal function, volume status, hemolysis, and thrombosis risk should be monitored, and ongoing need reassessed after immune recovery. No IVIG threshold, target, or schedule has been validated specifically for ABOi-KT.

## 10 Outcomes and competing harms

### 10.1 Survival benefit and residual early risk

Registry and center studies show that modern ABOi-KT provides excellent short- and long-term outcomes and can offer a survival advantage over waiting for a compatible deceased-donor kidney (3, 4). A multicenter analysis of 1,420 procedures demonstrated strong three-year outcomes across 101 centers (70), and long-running programs have reproduced favorable results (71). Meta-analyses nonetheless identify a modest early disadvantage relative to ABO-compatible living donation, driven particularly by ABMR, infection, and bleeding (5, 6). Counseling should therefore present ABOi-KT as a high-value route with additional early hazards, not as an immunologically identical transplant.

### 10.2 Infection, bleeding, and treatment burden are mechanistically linked

Infection risk reflects B-cell depletion, loss of protective immunoglobulins, maintenance exposure, and baseline vulnerability. Matched and multicenter studies have reported more urosepsis, opportunistic pneumonia, cytomegalovirus, or BK polyomavirus infection in ABOi recipients, although survival differences are not consistent (72, 73). Repeated TPE or DFPP can lower fibrinogen and other coagulation factors, while central access adds bleeding, thrombosis, and infection risk. National data also identify more early procedural complications (74). These harms are not incidental to desensitization; they are consequences of the immune compartments being manipulated and should be reported alongside antibody reduction.

### 10.3 Evidence remains vulnerable to selection and protocol heterogeneity

Most evidence is observational, and the apparent success of high-risk protocols partly reflects selection of recipients who reached transplantation. Assay methods, eligibility, induction, biopsy practice, rescue thresholds, and outcome definitions vary across centers. **Table 6** highlights representative studies that changed risk interpretation or protocol design; it should not be read as a rank ordering of regimens. Comparative effectiveness requires consistent reporting of both immunologic outcomes and competing harms.

**Table 6:** Representative evidence shaping contemporary risk interpretation.

Study	Design/cohort	Strategy or comparison	Practice-relevant finding
Barnett et al. (43)	2014; 67 ABOi-KT	Titer-stratified DFPP or IA; rituximab according to baseline titer	Tailored treatment was feasible and reduced unnecessary intervention; observational design
Opelz et al. (70)	2015; 1,420 recipients, 101 centers	ABO antibody reduction across heterogeneous programs	Strong 3-year outcomes at scale; substantial protocol heterogeneity
de Weerd et al. (6)	2018 meta-analysis; 1,346 ABOi and 4,943 controls	Multiple contemporary protocols	Good survival, but more early graft loss, ABMR, severe infection and bleeding than center-matched ABO-compatible controls
Scurt et al. (5)	2019 meta-analysis; 7,098 ABOi among 65,063 recipients	Mixed desensitization eras and strategies	Early outcome disadvantage diminished over longer follow-up; heterogeneity remained
Yin et al. (44)	2022; 100 living-donor ABOi-KT	Titer-based oral immunosuppression, antibody removal and rituximab escalation	Favorable 3-year outcomes supported individualized treatment in a Chinese center
Okada et al. (47)	2023; 142 low-titer recipients	100-mg rituximab versus rituximab-free desensitization	Acute ABMR 0% versus 7.6%; no clear infection increase in the rituximab group
Bleasel et al. (48)	2023; single-center cohort	No B-cell depletion in a contemporary protocol	Rituximab omission associated with more early acute rejection
Naciri Bennani et al. (66)	2024; 8 recipients with baseline titer ≥1:512	Rituximab, triple immunosuppression and specific/semi-specific apheresis	Median 13 sessions; 100% graft/patient survival, but 37% acute antibody-mediated rejection and frequent infection
Eum et al. (68)	2025; 271 ABOi-KT, 42 with titer ≥1:256	Plasmapheresis/IVIG intensified for high titers	Similar long-term survival; high-titer group required more treatment and had more chronic active ABMR
Mangin et al. (65)	2026 multicenter; 78 recipients with IgG ≤1:8	Pretransplant apheresis versus none	More rejection without apheresis; 3-year event-free survival 90% versus 79%
Reuter et al. (67)	2026 survey; 27 German centers	High/very high-titer practice	All centers used rituximab; IA predominated; eligibility and stopping criteria varied markedly

This table is selective and prioritizes studies that changed risk interpretation or protocol design. Cross-study comparisons are limited by assay, era, eligibility, and protocol heterogeneity.

## 11 Boundary conditions and future directions

### 11.1 Pediatric and deceased-donor pathways are biologically distinct

ABOi-KT is feasible in carefully selected children, including low-antibody protocols in younger recipients, but developmental immunology, small cohorts, and lifetime immunosuppressive exposure limit extrapolation from adults (75, 76). Deceased-donor ABO-incompatible pathways lack the elective preparation of living donation. A2/A2B donor allocation into B or O recipients also exploits lower antigen expression and should not be conflated with conventional A1/B-incompatible living-donor protocols.

### 11.2 Graft-directed blood-group conversion may change the problem

Enzymatic removal of A/B glycans from donor organs could shift intervention from the recipient immune system to the graft. In a human decedent model, transplantation of an enzyme-converted type-A kidney into a type-O recipient with high anti-A was not followed by hyperacute rejection and showed no ABMR for the first 2 days; from day 3, regeneration of endothelial A antigen coincided with complement deposition and antibody-mediated lesions, followed by higher Banff scores (77). Enzymatic conversion should therefore be viewed as a potentially temporary antigen-low window rather than permanent immunologic erasure. It may reduce the immediate antibody target long enough for cytoprotective endothelial programs to emerge, but it does not necessarily remove the need for accommodation. Indeed, the study detected increased expression of accommodation-related genes, although this finding is hypothesis-generating and does not establish durable protection. If A/B glycans reappear while circulating isoagglutinins remain, delayed ABMR is biologically plausible. Early clinical trials should serially assess tissue antigen re-expression where feasible, same-method anti-A/B titers, graft function, proteinuria, hemolysis/TMA markers, complement activation, and for-cause or protocol histology, with prespecified rescue criteria. They should also determine whether partial antibody lowering or transient complement control is still needed. Durability, conversion uniformity, manufacturing control, off-target tissue effects, and clinical safety require prospective evaluation.

### 11.3 Biomarkers should discriminate antibody quantity from pathogenic potential

The most useful next-generation assay will not merely reproduce an agglutination titer with greater automation. It should add information about complement activation, Fc-dependent effector potential, epitope density, subclass, avidity, or active antibody-secreting cell responses and should predict biopsy-defined injury beyond standard clinical variables (34, 35). Serial multi-omic or endothelial-response signals are promising research directions, but their incremental value, turnaround time, and transportability must be tested before they guide de-escalation. Even biologically informative assays should not be regarded as clinically ready until common calibrators, external quality assessment, inter-laboratory reproducibility, actionable turnaround time, acceptable cost, and prospective incremental utility have been demonstrated.

Study design should reflect the dynamic biology. Baseline-only sampling cannot separate removal, redistribution, renewed synthesis, and accommodation. Prospective cohorts should collect standardized specimens before treatment, immediately after selected apheresis sessions, at transplantation, during early rebound, and at the time of protocol or indication biopsy. Linking these samples to digital pathology, endothelial transcripts, complement products, and clinical outcomes could identify whether the same titer represents different immune states. External validation is essential because the purpose of a biomarker is transportable prediction, not retrospective explanation within one assay system.

### 11.4 Artificial intelligence should integrate—not substitute for—standardized immune phenotyping

Artificial intelligence and machine-learning methods could integrate longitudinal titer trajectories, IgM/IgG and subclass profiles, complement-binding activity, B-cell and plasmablast phenotypes, endothelial or transcriptomic signals, digital pathology, HLA data, immunosuppressive exposure, and clinical events. Multimodal risk systems in kidney transplantation and molecular classifiers for ABMR demonstrate the feasibility of combining clinical, histologic, immunologic, and transcriptomic information (78, 79). In ABOi-KT, clinically relevant targets would include prediction of antibody rebound, biopsy-defined injury, accommodation, and the marginal benefit of protocol de-escalation—not prediction of titer alone.

High-dimensional models may, however, amplify assay drift, center effects, missingness, class imbalance, treatment confounding, and temporal data leakage. Development should use prespecified clinical endpoints, longitudinal sampling, transparent feature definitions, calibration and decision-curve analysis, external validation across centers and assay platforms, and prospective evaluation of whether model-guided decisions improve outcomes. Explainability and algorithmic performance must be assessed alongside turnaround time, cost, workflow integration, fairness, privacy, and model updating; no algorithmic output should replace biopsy or multidisciplinary judgment until clinical utility has been demonstrated.

### 11.5 A research agenda for transplant antibody-mediated immunity

Priority studies should distinguish antibody abundance from pathogenic function and should sample the system dynamically. Standardized specimens obtained before treatment, after selected apheresis sessions, at transplantation, during rebound, and at biopsy could link IgM/IgG quantity, subclass, C1q binding, glycan fine specificity, B-cell/plasmablast states, complement products, endothelial transcripts, and clinicopathologic outcomes. Prospective multicenter validation is essential because the objective is transportable immune phenotyping, not increasingly elaborate center-specific thresholds.

## 12 Discussion and conclusions

ABO-incompatible kidney transplantation succeeds by controlling a changing antibody-mediated immune response, not by erasing a number. Natural anti-A/B antibodies differ in isotype, subclass, fine specificity, and effector potential; their production is distributed across memory B cells, plasmablasts, and long-lived plasma cells; and their pathogenic expression depends on complement, Fc-dependent cells, endothelial state, and coagulation. Accommodation adds a further, still incompletely defined layer: the term may encompass several states in which graft resistance and reduced effective antibody pathogenicity coexist in different proportions, without proving immunologic silence.

This compartmental model clarifies the limits of treatment. Apheresis changes the circulating pool, rituximab changes CD20-positive B-cell availability, anti-CD38 or proteasome inhibition changes antibody-secreting cells, and complement blockade changes downstream injury. A low titer cannot prove absence of memory or pathogenic antibody quality; B-cell depletion cannot prove plasma-cell control; and C4d cannot by itself distinguish accommodation from ABMR. Mapping a drug to an immune compartment establishes biological rationale, not ABO-specific clinical efficacy: direct evidence for anti-CD38 or proteasome-directed therapy remains case-level and should not be conflated with the more developed, but still heterogeneous, HLA desensitization and ABMR literature.

Clinical translation should emphasize proportionality. Recent multicenter low-titer evidence supports testing minimization prospectively rather than assuming that one low result permits omission of apheresis. Successful transplantation is feasible in selected recipients with high or very high titers, but this must be balanced against the cumulative burden of procedures, immune depletion, infection, bleeding, and the possibility that the transplant-day target will not be reached. Eligibility, readiness, modality-switch, toxicity, and stopping criteria should be defined before treatment begins.

This narrative Review is limited by heterogeneous assays, center-specific protocols, predominantly observational clinical evidence, and small mechanistic cohorts. The evidence asymmetry is particularly important for plasma-cell-directed therapy: ABO-specific support is confined to isolated rescue reports, often involving simultaneous ABO and HLA incompatibility and multiple concomitant treatments, whereas prospective or controlled studies address HLA sensitization or HLA-related ABMR. These antibody targets, populations, treatment contexts, and outcomes are not interchangeable. The Review therefore uses these data to define mechanistic plausibility and research priorities rather than to claim validated efficacy or a routine treatment algorithm for ABOi-KT.

The next advance should evaluate functional antibody phenotyping within standardized, prospective, multicenter frameworks linked to biopsy-defined outcomes. Routine clinical use should follow—not precede—demonstrated analytical reproducibility, actionable turnaround time, acceptable cost, and incremental utility beyond established clinicopathologic assessment. ABOi-KT is valuable not only as a route to living-donor transplantation, but also as a human model in which antibody production, vascular injury, and graft adaptation can be studied across time. That immunologic framing provides the strongest basis for precise, reproducible, and clinically proportionate intervention.
